# Physical activity and air pollution: context matters for cardiovascular health

**DOI:** 10.1016/j.lanwpc.2025.101612

**Published:** 2025-06-23

**Authors:** Omar Hahad, Sadeer Al-Kindi

**Affiliations:** a Department of Cardiology – Cardiology I, University Medical Center of the Johannes Gutenberg-University Mainz, Mainz, Germany; b German Center for Cardiovascular Research (DZHK), Partner Site Rhine-Main, Mainz, Germany; c Department of Cardiology, Houston Methodist, Houston, TX, USA; d Center for Health and Nature, Houston, TX, USA

Physical activity is strongly associated with improved cardiovascular, metabolic, and mental health.^1^ Meanwhile, long-term exposure to air pollution, especially fine particulate matter (PM_2.5_), is linked to higher risks of premature death, particularly from cardiovascular and respiratory diseases.^2^^,^^3^ Although both exert largely independent effects, concerns are growing about their interaction, especially in polluted environments where increased breathing during exercise may amplify pollutant inhalation and systemic exposure.^4^^,^^5^ Some studies suggest that air pollution may reduce the short-term benefits of physical activity for acute outcomes like asthma or heart rate variability, however, evidence for long-term outcomes is more mixed.^6^ A few studies suggest modest reductions in benefits at higher pollution levels, while others find no clear interaction. Nonetheless, public health models estimate that in most settings, especially for active commuting, the benefits of physical activity outweigh the risks of pollution exposure.^7^ Large-scale prospective studies exploring this interaction, particularly in low- and middle-income countries (LMICs), remain limited. In LMICs, pollution levels tend to be higher, and physical activity is more likely to occur through active commuting than structured leisure.

In *The Lancet Regional Health—Western Pacific,* Hao and colleagues^8^ analyze data from nearly 40,000 adults in the China substudy of the PURE (Prospective Urban Rural Epidemiology) cohort and examine long–term relationships between physical activity (self-reported), PM_2.5_ exposure (modelled annual ambient levels), and cardiovascular outcomes (non-fatal myocardial infarction, stroke, and heart failure or death from cardiovascular causes) and composite outcome (all-cause mortality + cardiovascular outcomes). Participants were followed for a median of nearly 12 years and stratified into high and low exposure groups based on a median PM_2.5_ level of 47.7 μg/m^3^. Higher total physical activity was associated with lower risk of a composite outcome of cardiovascular events and mortality (HR 0.84; 95% CI: 0.73–0.97) and major cardiovascular events (HR 0.80; 95% CI: 0.67–0.95) in the lower than median pollution areas. In high-pollution areas, these associations were not significant (HRs 0.97 and 0.98). Non-recreational activity, such as occupational or transport-related exertion, was associated with lower risk only in low-pollution areas but not in high-pollution areas. A non-statistically significant antagonistic interaction between high PM_2.5_ exposure and low non-recreational activity was also noted.

These findings align with known biological mechanisms. Exercise increases respiratory rate and pollutant uptake, which can trigger inflammation, oxidative stress, and vascular dysfunction^9^—pathways linked to cardiovascular disease.^10^ The study's focus on non-recreational activity is especially relevant for LMICs, where outdoor labor and commuting account for much of daily activity. In such settings, individuals may have little control over exposure. While recreational activity was not significantly associated with outcomes, low participation (∼54%) may have limited detection of effects.

The median PM_2.5_ level—nearly ten times the WHO-recommended limit of 5 μg/m^3^, underscores the high pollution burden in this population. These findings suggest that physical activity remains beneficial across pollution levels common in high-income countries, though benefits may be diminished in extreme settings. The attenuation of physical activity effect size at higher pollution is in line with prior studies. For example, a study from China showed that active commuting and farming activity was associated with higher cardiovascular risk in individuals exposed to very high pollution (PM_2.5_ ≥54 μg/m^3^).^11^ These studies raise important questions: Should people limit outdoor activity on high-pollution days? Can wearable air quality monitors guide behavior? How should outdoor workers in highly polluted areas be advised? Current global physical activity guidelines do not consider environmental exposures. This study highlights the need for more context-sensitive recommendations. Additionally, although the interaction between high pollution and high total activity was not statistically significant, the trend warrants further research to clarify its significance and potential health implications.

The findings of the study need to be examined within the context of its limitations. Physical activity was self-reported only at baseline and not updated over time. Pollution exposure was estimated at the community level, which may miss individual variation or short-term peaks. The study did not assess other pollutants or PM_2.5_ composition, both of which may influence outcomes. As an observational study, causality cannot be established, and reverse causality, where individuals with poorer health are less active, remains a possibility. Future studies should include comprehensive individual-level exposure data (e.g. PM_2.5_, temperature, etc.), objective activity measures like accelerometers, and account for seasonal or short-term variability. Policies to promote physical activity should be paired with efforts to improve air quality and urban design solutions, such as green spaces and clean-air corridors, could provide dual benefits for health and sustainability.

In conclusion, this study provides valuable evidence that environmental conditions may influence the relationship between physical activity and cardiovascular risk. While physical activity is generally associated with improved health, its benefits may vary by environmental context. Promoting physical activity and clean air must go hand in hand to maximize public health impact.

## Contributors

OH conceptualized the manuscript and wrote the first draft. SA-K contributed to the interpretation of the literature and critically revised the manuscript. Both authors approved the final version.

## Declaration of interests

None.
